# Whole Body *Ip6k1* Deletion Protects Mice from Age-Induced Weight Gain, Insulin Resistance and Metabolic Dysfunction

**DOI:** 10.3390/ijms23042059

**Published:** 2022-02-12

**Authors:** Sarbani Ghoshal, Sandip Mukherjee, Molee Chakraborty, Eliwaza Naomi Msengi, Jake Haubner, Anutosh Chakraborty

**Affiliations:** 1 Department of Metabolism and Aging, The Scripps Research Institute, Jupiter, FL 33458, USA; 2 Department of Biological Sciences and Geology, QCC-City University of New York, New York, NY 11364, USA; 3 Department of Pharmacology and Physiology, Saint Louis University School of Medicine, Saint Louis, MO 63104, USA; sandip.mukherjee@health.slu.edu (S.M.); molee.chakraborty@health.slu.edu (M.C.); eliwazanao.msengi@slu.edu (E.N.M.); jake.haubner@slu.edu (J.H.)

**Keywords:** IP6K1, aging, metabolism, adipose tissue browning, weight gain, insulin resistance

## Abstract

(1) Background: We previously demonstrated that disruption of IP6K1 improves metabolism, protecting mice from high-fat diet-induced obesity, insulin resistance, and non-alcoholic fatty liver disease and steatohepatitis. Age-induced metabolic dysfunction is a major risk factor for metabolic diseases. The involvement of IP6K1 in this process is unknown. (2) Methods: Here, we compared body and fat mass, insulin sensitivity, energy expenditure and serum-, adipose tissue- and liver-metabolic parameters of chow-fed, aged, wild type (*aWT*) and whole body *Ip6k1* knockout (*aKO*) mice. (3) Results: IP6K1 was upregulated in the adipose tissue and liver of *aWT* mice compared to young *WT* mice. Moreover, *Ip6k1* deletion blocked age-induced increase in body- and fat-weight and insulin resistance in mice. *aKO* mice oxidized carbohydrates more efficiently. The knockouts displayed reduced levels of serum insulin, triglycerides, and non-esterified fatty acids. *Ip6k1* deletion partly protected age-induced decline of the thermogenic uncoupling protein UCP1 in inguinal white adipose tissue. Targets inhibited by IP6K1 activity such as the insulin sensitivity- and energy expenditure-inducing protein kinases, protein kinase B (PKB/Akt) and AMP-activated protein kinase (AMPK), were activated in the adipose tissue and liver of *aKO* mice. (4) Conclusions: *Ip6k1* deletion maintains healthy metabolism in aging and thus, targeting this kinase may delay the development of age-induced metabolic dysfunction.

## 1. Introduction

The increased prevalence of obesity is a major health concern. Aging significantly increases the risks of obesity and obesity-induced co-morbidities such as type-2 diabetes mellitus (T2DM), non-alcoholic fatty liver disease/steatohepatitis (NAFLD/NASH), cardiovascular disease and certain types of cancer [1,2,3,4]. Excess fat accumulation exacerbates frailty in older persons. Conversely, metabolic dysfunction accelerates aging, as young people with obesity and T2DM often exhibit features of accelerated aging [5].

Aging impairs functions of metabolic tissues such as adipose tissue, liver, and skeletal muscle [5,6,7]. In young and healthy conditions, the white adipose tissue (WAT) stores excess energy as triglycerides (TAG). Conversely, the mitochondria-enriched adipocytes in the brown adipose tissue (BAT) and brown-like “beige” or “brite” adipocytes in certain WAT depots (for example, the inguinal depot or IWAT) express mitochondrial uncoupling protein 1 (UCP1) to expend energy by thermogenesis. This process facilitates fat loss and improves insulin sensitivity in rodents and humans [8,9,10,11,12]. Moreover, adipose tissue is a major endocrine organ, secreting numerous adipokines that regulate energy metabolism, inflammation, and various other processes in the body [13]. Chronic energy accumulation in obesity and/or aging disrupts energy homeostasis, alters adipokine secretion and immune response, causing adipose tissue dysfunction [14,15]. Dysfunctional adipocytes release inflammatory cytokines that reduce their fat-storing ability and increase the levels of non-esterified fatty acid (NEFA), which causes insulin resistance and lipotoxic liver injury [16,17]. Age-induced depletion of brown/beige adipocytes and subsequent loss of UCP1 have been shown to aggravate metabolic pathologies in rodents and humans [5,18,19,20]. Moreover, obesity- and/or aging-induced hyperinsulinemia causes pathway-selective insulin resistance in the liver, reducing glucose uptake and increasing gluconeogenesis and de novo lipogenesis, which subsequently leads to hyperglycemia and lipotoxic liver injury [6,16,17,21,22].

The characterization of novel proteins and/or pathways involved in metabolism and aging is crucial to understand the mechanisms that regulate these processes, and to develop therapeutic strategies to improve healthspan [6,23]. The use of new mouse models that lack or overexpress a novel gene of interest is particularly useful in this regard. Using various knockout mouse models, we previously discovered the inositol pyrophosphate (5-IP7) biosynthetic enzyme inositol hexakisphosphate kinase 1 (IP6K1) as a novel target in high-fat diet-induced obesity. A family of three IP6Ks synthesizes the biomolecule 5-IP7 in mammals by phosphorylating inositol hexakisphosphate (IP6) [24,25,26,27,28]. IP6K1 regulates metabolic functions of adipose tissue, liver and pancreatic β cells, whereas IP6K3 modulates metabolism of the skeletal muscle [26,29,30,31,32]. IP6K1 impairs insulin signaling by inhibiting the insulin effector protein kinase Akt, promoting high-fat diet-induced insulin resistance [33]. Moreover, IP6K1 reduces whole-body energy expenditure by inhibiting adipose tissue browning and thermogenesis [34]. IP6K1 diminishes energy expenditure and stimulates fatty acid biosynthesis by inhibiting the metabolism enhancing AMP-activated protein kinase (AMPK) [34,35]. IP6K1 also reduces serum levels of the insulin-sensitizing and metabolically favorable adipokine, adiponectin [34,35]. Consequently, high-fat diet-fed, whole-body- or adipocyte-specific *Ip6k1-KO* mice are protected from obesity, hyperinsulinemia, insulin resistance, and hepatic steatosis [33,34,35]. Moreover, whole-body- or hepatocyte-specific *Ip6k1* deletion protects mice from high-fat and high-cholesterol (Western) diet-induced lipotoxic liver injury and NAFLD/NASH [36]. IP6K1 also promotes insulin secretion from pancreatic β cells [31,32,37]. Accordingly, *Ip6k1* deletion diminishes insulin secretion whereas transgenic mice that express a hyperactive IP6K1 display augmented insulin release, congenital hyperinsulinemia, and obesity [32]. In summary, IP6K1 regulates metabolism via pleiotropic mechanisms.

High-fat diet-induced obesity in rodents does not exactly mimic human obesity. A major reason is that such high percentages of fat (40–60% kcals from fat) are not regularly consumed by humans. Conversely, aged mice develop metabolic dysfunction despite consuming a normal chow diet (~62%, ~25% and ~13% kcals, from carbohydrate, protein, and fat, respectively). Mechanistically, high-fat diet-induced obesity is caused due to rapid accumulation of fat, whereas aging-induced weight gain occurs because of fat synthesis from carbohydrates over time. Thus, determining the impact of a protein on both models of metabolic dysfunction is necessary. However, no studies have yet been done to define IP6K1’s role in aging-induced metabolic dysfunction. To address this, here we compared metabolic features of chow-fed, aged (22-months), *WT* and *Ip6k1-KO* (*aWT* and *aKO*) mice. Female subjects are often overlooked in research, including metabolic research, despite the sex-specific differences in phenotypes [38,39,40,41]. Therefore, in this study, we used mice of both sexes.

## 2. Results

### 2.1. Body Weight and Composition

#### Whole-Body Deletion of *Ip6k1* Protected Mice from Age-Induced Weight and Fat Gain

**Figure 1:**
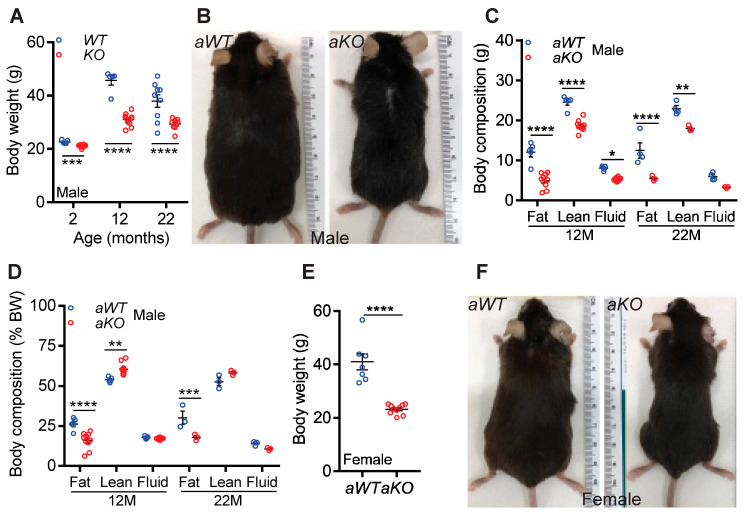
Whole-body deletion of *Ip6k1* protected mice from age-induced weight and fat gain. Whole-body deletion of *Ip6k1* protected mice from age-induced weight and fat gain. (**A**,**B**). *Ip6k1* deleted (*KO*) male mice were substantially protected from age-induced increase in body weight. *n* = 5 and 10 for 2- and 12-month-old *WT* and *KO*; and 9 each for 22-month-old mice). (**C**). Total body fat, lean and fluid mass were significantly reduced in *aKO* male mice. (**D**) Percent fat mass (normalized to total body weight) was substantially less in *aKO* male mice. Percent lean mass was higher in 12M-old *aKO*, whereas its was similar in 22M-old *aKO* and *aWT* male mice. Percent fluid mass was similar in both genotypes. *n* = 5 and 10 for 12-month-old *WT* and *KO*; and 3 each for 22-month-old male mice. (**E**,**F**) *aKO* (22-month-old) female mice were protected from age-induced increase in body weight. *n* = 7 and 10 for *aWT* and *aKO* female mice. Number of mice (*n*) used in each experiment are presented as individual datapoints. Mean ± s.e.m. values are shown within dot plots. For 2 independent data sets, two-tailed unpaired Student’s *t*-test was used. * *p* < 0.05, ** *p* < 0.01, *** *p* < 0.001, **** *p* < 0.0001.

We determined whether aging differentially regulated body weight and composition in *WT* and *KO* male and female mice. Young, chow-fed *KO* male mice displayed a slight, albeit significant reduction in body weight (Figure 1A), which conforms with previous reports [33,37]. Interestingly, with age, *WT* males gained substantially more body weight than *KO* males (Figure 1A,B). Body composition analysis revealed that total fat mass was considerably lower in 12- and 22-month-old *aKO,* compared to *aWT* males (Figure 1C). Total lean and fluid mass were also reduced in *aKO* males, although to a lesser extent than fat mass (Figure 1C). The percent fat mass (normalized to total body weight) was also significantly reduced in 12- and 22-month-old *aKO* male mice (Figure 1D). Percent lean mass increased in 12-month-old *aKO* and was similar in 22-month-old *aWT* and *aKO* male mice (Figure 1D). Percent fluid mass was similar in both genotypes.

Aged (22-month-old) female *aKO* mice displayed an even greater difference in body weight, compared to *aWT* mice (Figure 1E,F). Like males, both total and percent fat mass were substantially lower in female *aKO* mice (Figure S1A,B). Total lean mass decreased but percent lean mass increased in female *aKO* mice (Figure S1A,B). Fluid mass was marginally reduced in *aKO* females. Thus, *aKO* mice displayed an overall reduction in body size. However, when normalized to body weight, *Ip6k1* deletion specifically blocked aging-induced accumulation of fat without significantly altering lean or fluid mass.

### 2.2. Serum Metabolic Profiles

#### *aKO* Mice Displayed Improved Serum Metabolic Profiles

**Figure 2:**
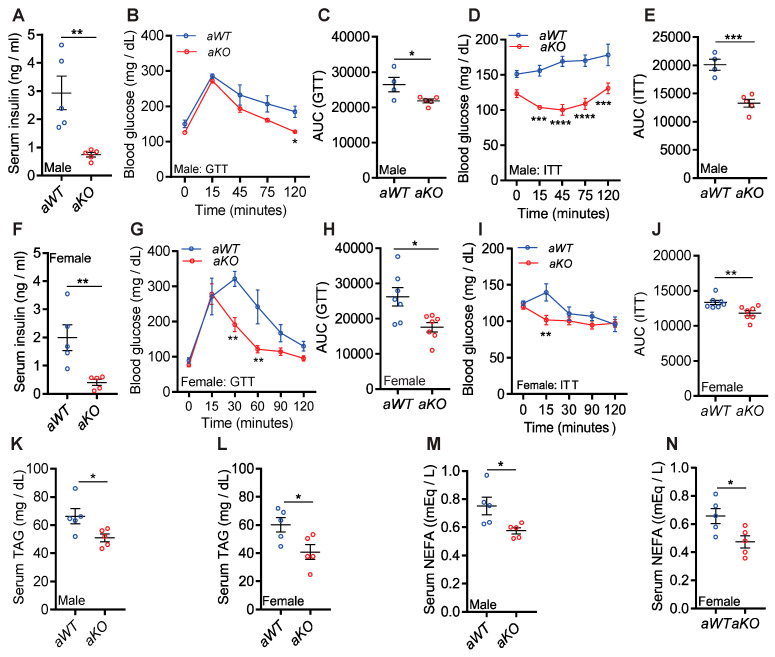
*aKO* mice displayed improved serum metabolic profiles. (**A**) *aKO* male mice were protected from aging-induced hyperinsulinemia. *n* = 5 mice per group. (**B**,**C**) Male *aKO* mice disposed blood glucose more efficiently than *aWT* mice following exogenous glucose injection (glucose tolerance test—GTT). The assay was performed in 15-month-old mice. *n* = 4 and 5 for *aWT* and *aKO* mice, respectively. (**D**,**E**) *aKO* but not *aWT* male mice efficiently disposed blood glucose following exogenous insulin injection (insulin tolerance test–ITT). The assay was performed in 16-month-old mice. *n* = 4 and 5 for *aWT* and *aKO* mice, respectively. (**F**) Female *aKO* mice were protected from hyperinsulinemia. *n* = 5 mice each group. (**G**,**H**) Female *aKO* mice displayed substantially improved glycemic profiles in a GTT. The assay was performed in 15-month-old mice. *n* = 7 mice per group. (**I**,**J**) Female *aKO* mice showed slightly improved glucose disposal in an ITT. The assay was performed in 16-month-old mice. *n* = 7 mice per group. (**K**,**L**) Serum TAG levels were reduced in *aKO* male and female mice compared to *aWT* littermates. *n* = 5 mice each group. (**M**,**N**) Both sexes of *aKO* mice displayed reduced serum NEFA levels. *n* = 5 mice each group. Number of mice (*n*) used in each experiment are presented as individual datapoints. Mean ± s.e.m. is shown within dot plots. For multiple comparisons, two-way ANOVA with Holm-Šidák multiple comparison test and for 2 independent data sets, two-tailed unpaired Student’s *t*-test were used. * p < 0.05, **p < 0.01, *** *p* < 0.001, **** *p* < 0.0001.

Next, we checked whether the lean phenotype delayed age-induced metabolic aberration in *aKO* male and female mice. *aWT* and *aKO* male mice displayed average serum insulin levels of 2.9 and 0.74 ng/mL, respectively (Figure 2A). These values were 1.2 and 0.4 ng/mL in young *WT* and *KO* mice [37]. Thus, *aKO* mice maintained normal levels of serum insulin. Moreover, ad libitum blood glucose levels were higher in *aWT* but not in *aKO* mice (Figure S2A) (normal value ~120 mg/dL). Next, we tested the efficiency of glucose disposal in *aWT* and *aKO* male mice in a glucose tolerance test (GTT). Although fasting (16 h) blood glucose levels are similar in young *WT* and *KO* mice (~70 mg/dL) [37], aging-induced increase in fasting blood glucose levels was less in *aKO* mice (average values were 126 and 150 mg/dL in *aKO* and *aWT* mice, respectively; 0 time point of GTT; *p* = 0.0544; Figure 2B). Moreover, *aKO* male mice disposed of blood glucose more efficiently than *aWT* in a glucose tolerance test (GTT) (Figure 2B,C). Both young *WT* and *KO* male mice efficiently disposed of blood glucose following exogenous insulin injection (insulin tolerance test—ITT) [37]. However, *aKO* but not *aWT* male mice showed improved glucose disposal in an ITT (Figure 2D,E).

Ad libitum blood glucose levels were similar in *aWT* and *aKO* female mice (data not shown). Yet, female *aKO* mice showed reduced serum insulin and improved glycemic profiles in a GTT (Figure 2F–H). Exogenous insulin injection reduced blood glucose in both female genotypes, albeit slightly more efficiently in *aKO* mice (Figure 2I,J). Different glycemic profiles in *aWT* male and female mice in ITT indicated that aged males were more insulin resistant than female mice, which is a commonly observed phenomenon in mice [42].

Aging did not increase serum TAG levels in *WT* (compared to reported values in young *WT* [43]). These values were lower in *aKO*, compared to *aWT* mice (Figure 2K,L). Serum NEFA levels were higher in aged mice of both genotypes, compared to young mice [43]. However, the increase was less pronounced in *aKO* mice, and thus *aKOs* displayed reduced serum NEFA levels than *aWT* mice (Figure 2M,N). Serum cholesterol and phospholipid levels were largely similar (Figure S2B–E). These results suggest improved serum metabolic profiles in *aKO* mice.

### 2.3. Energy Expenditure, Food Intake and Activity

#### *aKO* Mice Expended Carbohydrates More Efficiently Than *aWT* Mice

**Figure 3:**
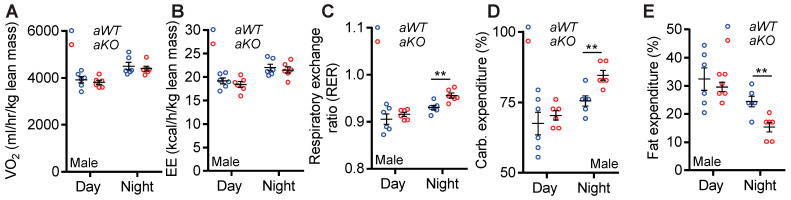
*aKO* mice expended carbohydrates more efficiently than *aWT* mice. (**A**) *aWT* and *aKO* mice consumed oxygen (VO2) to a similar extent. (**B**) Average energy expenditure (EE) was similar in *aWT* and *aKO* mice. (**C**) The average nighttime RER value was significantly higher in *aKO* mice. (**D**) *aKO* mice efficiently expended carbohydrate during nighttime. (**E**) During nighttime, *aKO* mice oxidized less fat than *aWT* mice. *n* = 6 mice each group. Number of mice (*n*) used in each experiment are presented as individual datapoints. Mean ± s.e.m. values are shown within dot plots. For 2 independent data sets, two-tailed unpaired Student’s *t*-test was used. ** *p* < 0.01.

Like chow- and high-fat-fed young mice [35], *aWT* and *aKO* mice consumed similar amounts of food (Figure S3A,B). When normalized to body weight, *aKO* mice exhibited a slight but significant increase in food intake (Figure S3C,D). Slight increase in food intake was presumably necessary to compensate for the negative energy balance in the *aKO* mice. Total and ambulatory activity profiles were largely similar in both genotypes (Figure S3E,F). Whole body energy expenditure studies showed that the volume of oxygen consumption (VO_2_) was similar in both aged genotypes (Figure 3A). Accordingly, energy expenditure (EE) was also similar in *aWT* and *aKO* mice (Figure 3B). However, the nighttime respiratory exchange ratio (RER) was significantly higher in the *aKO* mice (Figure 3C). Thus, *aKO* expended more carbohydrate and less fat than *aWT* mice during nighttime (Figure 3D,E). The major source of energy in the chow-diet is carbohydrate. Thus, age-induced weight gain in mice is largely due to increased lipogenesis rather than augmented accumulation of exogenous fat, which is observed in high-fat diet-induced obesity. Efficient carbohydrate oxidation reduced fat synthesis and accumulation, leading to leanness, and metabolic improvements in chow-fed *aKO* mice.

### 2.4. Metabolic Parameters of Adipose Tissue and Liver

#### Age-Induced Metabolic Aberration in Adipose Tissue and Liver Was Ameliorated in *Ip6k1* Deleted Mice

**Figure 4:**
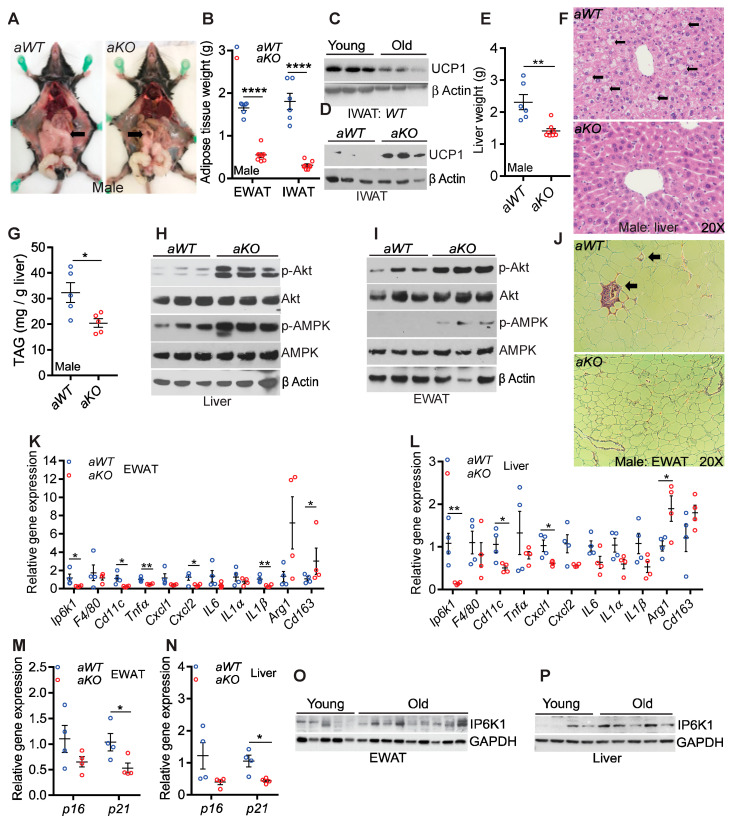
Age-induced metabolic aberration in adipose tissue and liver was ameliorated in Ip6k1 deleted mice. (**A**) *aKO* male mice displayed reduced fat accumulation in adipose tissue depots. (**B**) Weight of epididymal and inguinal (EWAT and IWAT) adipose tissue depots was reduced in male *aKO* mice. *n* = 6, and 8 for *aWT* and *aKO* mice. (**C**) Aging downregulated UCP1 protein in the IWAT of *WT* mice. (**D**) UCP1 protein levels were higher in the IWAT of *aKO*, compared to *aWT* mice. (**E**) Liver weight was reduced in *aKO* mice. *n* = 6, and 8 for *aWT* and *aKO* mice. (**F**,**G**) *aKO* mice exhibited reduced micro-steatosis and TAG accumulation in the liver. *n* = 5 mice per group. (**H**,**I**) Stimulatory phosphorylation of Akt (S473) and AMPK (T172) increased in the liver and EWAT of *aKO* mice. (**J**) Adipocyte size (EWAT) was reduced and crown like structures (indicated by an arrow) were absent in the EWAT of *aKO* mice. (**K**) mRNA expression of *Cd11c*, *Tnfα*, *IL1β* and *Cxcl2* were reduced and *Cd163* increased in the EWAT of *aKO* mice. *IL6*, *IL1α*, *Cxcl1* and *Arg1* were insignificantly altered. F4/80 expression was similar in *aWT* and *aKO* EWAT. *Ip6k1* expression was determined to confirm the genotypes. *n* = 4 mice per group. (**L**) mRNA expression of *Cd11c* and *Cxcl1* were diminished whereas *Arg1* was augmented in the liver of *aKO* mice. *Tnfα*, *Cxcl2*, *IL6*, *IL1α*, *IL1β* were downregulated and *Cd163* was upregulated to insignificant levels. *F4/80* expression was similar in *aWT* and *aKO* liver. *n* = 4 mice per group. (**M**,**N**) mRNA expression of *p21* was significantly reduced in EWAT and liver of *aKO* compared to *aWT* controls. *p16* was also downregulated in *aKO* tissues albeit to insignificant levels. *n* = 4 mice per group. (**O**,**P**) Aging upregulated IP6K1 at the protein level in EWAT and liver tissues of *WT* mice. *n* = 4 and 5 and 5 and 7 per group for EWAT and liver, respectively. Number of mice (*n*) used in each experiment are presented as individual datapoints. Mean ± s.e.m. values are shown within dot plots. For 2 independent data sets, two-tailed unpaired Student’s *t*-test was used. * *p* < 0.05, ** *p* < 0.01, **** *p* < 0.0001.

In high-fat diet-fed mice, IP6K1 promotes obesity and insulin resistance by reducing insulin signaling and thermogenic energy metabolism in adipose tissue and liver [33,34,36]. Here, we checked whether these parameters were improved in *aKO* mice. *aKO* mice accumulated substantially less fat in epididymal (gonadal in females) and inguinal adipose tissue depots (EWAT/GWAT and IWAT) (Figure 4A and Figure S4A, GWAT indicated by arrows, Figure 4B and Figure S4B). UCP1, which is known to be downregulated in aging [5,18,19,20], was reduced in the IWAT of *aWT*, compared to young *WT* mice (Figure 4C and Figure S4C). Interestingly, UCP1 expression was higher in the IWAT of *aKO* compared to *aWT*, indicating that the browning property was partly maintained in the aged IWAT following *Ip6k1* deletion (Figure 4D). UCP1 levels in the BAT was similar in *aWT* and *aKO* mice (data not shown), which is explainable as IP6K1 regulates browning of WAT without altering BAT functions [34]. Like adipose tissue, liver weight was also reduced in male and female *aKO* mice (Figure 4E and Figure S4D). Microsteatosis developed to a much higher extent in *aWT* compared to *aKO* mice (Figure 4F and Figure S4E, indicated by arrows). Accordingly, *aKO* mice accumulated less TAG in the liver (Figure 4G). IP6K1 diminishes insulin signaling and energy expenditure by inhibiting the protein kinases Akt and AMPK [33,34,35]. Accordingly, stimulatory phosphorylation levels of Akt (S473) and AMPK (T172) increased in the adipose tissue and liver of *aKO* mice (Figure 4H,I and Figure S4F–I).

Histology of EWAT showed reduced adipocyte size in *aKO* mice (Figure 4J). Obesity- or aging-induced adipocyte dysfunction triggers infiltration of inflammatory M1 macrophages and reduces the population of insulin-sensitizing M2 macrophages [44,45,46,47,48]. Infiltrated macrophages form the crown-like structures in the adipose tissue, which was present in *aWT* but not in *aKO* mice (Figure 4J, indicated by arrows). Moreover, markers of M1 macrophages such as *Cd11c*, *Tnfα*, *IL1β* and *Cxcl2* were downregulated whereas the M2 marker *Cd163* was upregulated in the EWAT of *aKO* mice (Figure 4K). Other inflammatory markers *IL6*, *IL1α* and *Cxcl1* and the M2 marker *Arg1* were similarly but insignificantly altered. Expression levels of *F4/80* were unaltered, indicating that the total population of macrophages was similar in the EWAT of *aWT* and *aKO* mice (Figure 4K). Like EWAT, markers of the M1 and M2 macrophages were altered in the liver of *aKO* mice (Figure 4L). Aging did not substantially increase serum levels of the hepatotoxicity markers aspartate aminotransferase (AST) and alanine aminotransferase (ALT) in *aWT* mice. Therefore, levels of these markers were largely comparable between genotypes with a slight reduction in AST in the female *aKO* mice (Figure S4J–M).

Improved metabolism and reduced inflammation may have delayed senescence in the adipose tissue and liver of *aKO* mice, as the senescent markers *p16* and *p21* were downregulated in the knockouts (Figure 4M,N). Finally, we determined whether IP6K1 is an age-inducible protein in adipose tissue and liver. Aging upregulated IP6K1 protein but not its mRNA expression in these tissues (Figure 4O,P and Figure S4N–P).

Although 22-month-old *WT* mice, used in this study, developed metabolic dysfunction, we did not observe visual aging phenotypes in these mice. Compared to young *WT* and *KO* [35], *aWT* and *aKO* mice displayed slightly reduced activity levels, although no significant differences between genotypes were observed (Figure S3E,F). Skin texture and hair quantity and quality appeared normal and were similar in both aged genotypes (Figure 1B,F). Neither genotype developed cataract, kyphosis, or tumor. For senescence and survival studies, 28–36-month-old mice are recommended [49]. Therefore, whether improved metabolism in *Ip6k1*-deleted mice delays senescence and increases lifespan will be tested in 36-month-old mice in future studies.

## 3. Discussion

Age-induced metabolic dysfunction of adipose tissue and liver increases weight and fat gain and insulin resistance [5,19,20,50,51]. The current study shows that whole-body deletion of *Ip6k1* protects mice from age-induced weight gain, insulin resistance and metabolic dysfunction by improving metabolic functions of these tissues. Although both male and female *aKO* mice displayed overall metabolic improvements, the difference in glycemic profiles was more robust in male than female *aKO* mice compared to their respective *aWT* controls. This is explainable as male C57BL6 mice attain insulin resistance faster than female mice with age [42].

Adipocyte- or hepatocyte-specific *Ip6k1* deletion has been shown to improve metabolism and insulin sensitivity in high-fat, high-cholesterol or high-fructose diet-fed mice [34,36]. Here, we observed improved serum, adipose tissue and liver metabolic parameters and increased activities of the IP6K1 targets Akt and AMPK in these tissues of *aKO* mice. IP6K1 also promotes insulin secretion from the pancreatic β cells [31,32]. Thus, improved metabolic functions of these tissues contribute to the observed phenotypes in *aKO* mice. However, IP6K1 is ubiquitously expressed, including in the brain [26,52,53], and therefore the involvement of the hypothalamic-IP6K1 in metabolic regulation cannot be ruled out. Hypothalamic-AMPK enhances food intake [54]. Thus, a slight increase in food intake (when normalized to body weight) in *aKO* mice could be due to augmented AMPK activity in the hypothalamus.

Despite increased UCP1 expression and AMPK activation, overall energy expenditure was not increased in *aKO* mice. Although the reason is not entirely clear at this point, substantial reduction in body fat over time may have readjusted the metabolic rate in *aKO* mice. However, carbohydrate oxidation was higher, which explains reduced synthesis and accumulation of fat in the aged knockouts. This result is in line with our previous findings that young, chow-fed *Ip6k1*-*KO* mice exhibit higher carbohydrate oxidation, and IP6K1 reduces fatty acid biosynthesis in adipocytes [35]. Prolonged increase or decrease in energy expenditure may have negative effects on healthspan [50]. Efficient carbohydrate use may provide an alternative therapeutic strategy to ameliorate metabolic dysfunction in aging. Other IP6K1-regulated processes may also contribute to age-induced metabolic dysfunction. For example, IP6K1-generated 5-IP7 maintains the cellular polyphosphate levels and pyrophosphorylates protein targets [55,56] that regulate glycolysis, chemotaxis and phagosomal motility of macrophages [57,58,59]. Moreover, IP6K1, in coordination with another inositol pyrophosphate biosynthetic enzyme, PPIP5K, generates IP8 [60], which has also been shown to regulate energy metabolism [61]. Therefore, the observed metabolic phenotypes in *aKO* mice may also arise due to a reduction in IP8.

Age-induced upregulation of IP6K1 explains why IP7 levels are higher in cells isolated from older compared to younger mice [33,62]. Interestingly, protein but not mRNA levels of IP6K1 increased in aged adipose tissue, indicating involvement of post-translational events in IP6K1’s upregulation. IP6K2 is degraded via the ubiquitin-proteasomal system [63]. Future studies will determine whether IP6K1’s stability is regulated by similar mechanisms. Moreover, IP6K1 is phosphorylated at serine residues 118 and 121, which regulates its catalytic activity and interaction with metabolic proteins [32,64]. Whether aging induces IP6K1′s activity and protein-protein interaction via these mechanisms will be tested.

Among other *Ip6k* isoforms, *Ip6k2* is ubiquitously expressed, and its deletion decreases metastasis [65]. *Ip6k3* expression is limited to skeletal muscle and heart, and its whole-body deletion improves skeletal muscle metabolism, protecting mice from age-induced fat gain, insulin resistance and mortality [30]. Thus, targeting the IP6K pathway in general may improve healthspan. Encouragingly, pharmacologic inhibition of this pathway has been shown to ameliorate diet-induced obesity, insulin resistance, hepatic steatosis, osteoporosis, chronic kidney disease, ischemia/reperfusion injury, and myocardial infarction in mouse models [66,67,68,69,70]. Efforts are ongoing to develop potent and improved drug-like IP6K inhibitor compounds for clinical trials [71,72,73]. IP6K1 is relevant in human metabolic diseases as its levels positively correlate with HOMA-IR (Homeostatic Model Assessment for Insulin Resistance) in prediabetic subjects and negatively corelate to insulin sensitivity [74]. Moreover, IP6K1 is upregulated in the liver of NASH, cirrhosis, and hepatocellular carcinoma patients [36,75]. Hopefully, inhibiting IP6K1 or the IP6K pathway will delay age-induced metabolic dysfunction in humans.

## 4. Materials and Methods

### 4.1. Chemicals and Reagents

Unless otherwise stated, all the chemicals were purchased from Sigma/EMD Millipore, St. Louis, MO, USA. Antibodies—IP6K1 (HPA040825), UCP1 (U6382), β-actin (A5316) and GAPDH (G8795)—were from Sigma/EMD Millipore, St. Louis, MO, USA; p-AMPK (2535), AMPK (2793), p-Akt (4060) were from Cell Signaling Technology, Danvers, MA, USA; Total Akt (Sc-81434) were from Santa Cruz Biotechnology, Dallas, TX, USA. The insulin ELISA kit (90080) was from Crystal Chem Inc., Elk Grove Village, IL, USA. ALT, AST, and TAG assay kits were from Teco Diagnostics, Anaheim, CA, USA; insulin assay kit was from Insulin was from Novo Nordisk, Bagsværd, Denmark.
**qRT-PCR Primers (Forward and reverse).****Gene****Sequence**Ip6k1F: 5-TGGAAGTGGGGCAGTATGG-3 R: 5-CGTCGTACCGCATCATGCT-3F4/80F: 5-GGATATGGAAACTTCAACTGCAA-3 R: 5-CAAGTGTACAGAAGGAAGCATAAC-3CD11cF: 5-CAAATAGGTGGCCTCTACAAATG-3 R: 5-GTAGGACCACAAGCCAACA-3TNFαF: 5-AGACCCTCACACTCAGATCA-3 R: 5-GAGTAGACAAGGTACAACCCATC-3Cxcl1F: 5-ACCGAAGTCATAGCCACACTC-3 R: 5-CTCCGTTACTTGGGGACACC-3Cxcl2F: 5-CCCAGACAGAAGTCATAGCCAC-3 R: 5-TGGTTCTTCCGTTGAGGGAC-3IL6F: 5-TGAGAAAAGAGTTGTGCAATGG-3 R: 5-GGTACTCCAGAAGACCAGAGG-3IL1αF: 5-AGGGAGTCAACTCATTGGCG-3 R: 5-TGGCAGAACTGTAGTCTTCGT-3IL1βF: 5-TGCCACCTTTTGACAGTGATG-3 R: 5-TGATGTGCTGCTGCGAGATT-3Arg1F: 5-TTAGAGATTATCGGAGCGCCT-3 R: 5-GTCTCTCACGTCATACTCTGTTTCT-3Cd163F: 5-ATTCAGCGACTTACAGTTTCCTC-3 R: 5-ACAAAGATGTCAGTCCATCATCA-3P16F: 5-ATGGGTCGCAGGTTCTTGG-3 R: 5-TGCCCATCATCATCACCTGG-3P21F: 5-TTGCCAGCAGAATAAAAGGTGCC-3 R: 5-GACGAAGTCAAAGTTCCACCGT-3Hprt1F: 5-CAAACTTTGCTTTCCCTGGT-3 R: 5-TCTGGCCTGTATCCAACACTTC-3Rplp0F: 5-AGATTCGGGATATGCTGTTGGC-3 R: 5-TCGGGTCCTAGACCAGTGTTC-3

### 4.2. Animals

Animal care and experiments were approved by the Saint Louis University School of Medicine and The Scripps Research Institute Institutional Animal Care and Use Committee (IACUC). Male *WT* and *Ip6k1-KO* (*KO*) mice on pure C57BL6 background were housed in a 12 h light/12 h dark cycles at 23 °C and were fed a standard chow diet (Harlan Laboratories # 2018SX). At indicated time periods, body weight and composition, energy expenditure, blood glucose level, GTT and ITT were performed. After 22-months, mice were fasted for 4 h (to minimize nibbling induced changes in metabolism and signaling) and then euthanized by carbon dioxide asphyxiation. Tissues were processed for downstream applications. To compare mRNA and protein expression in young vs. old mice, 2- and 22-month-old *WT* mice were used.

### 4.3. Body Composition Analyses by Q-NMR

Fat, lean and fluid masses of mice were measured using the Minispec LF-NMR (Bruker Optics, Ettlingen, Germany) analyzer. Percent body composition was calculated based on the total body weight of mice.

### 4.4. Ad Libitum Glucose Level and Glucose and Insulin Tolerance Tests (GTT and ITT)

Ad libitum glucose, GTT and ITT were performed in 14-, 15- and 16-month-old mice following previously published procedures. For GTT, glucose (2 g/kg BW, i.p.) was injected in 16-h-fasted animals. For ITT, insulin (0.75 U/kg BW, i.p.) was injected in 5h-fasted mice. Blood glucose levels were measured by glucometer by puncturing tail veins of mice before and after the indicated time periods of injection [33,36,66].

### 4.5. Energy Expenditure and Locomotor Activity Studies

Mice were placed individually in metabolic cages with a precise thermostatic control in a Comprehensive Laboratory Monitoring System (CLAMS; Columbus Instruments, Columbus, OH, USA) and were acclimatized for 36 h. Afterwards, oxygen consumption, (VO_2_), carbon dioxide release (VCO_2_) and spontaneous locomotor activity were measured for 48 h, following standard procedures [66,76]. Respiratory exchange ratio (RER) and energy expenditure (EE) were calculated using the following equations: RER = VCO_2_/VO_2_; EE (kcal/h) = (3.815 + 1.232 × RER) × VO_2_. Values were normalized by lean body mass. Percent carbohydrate and fat expenditure was calculated following standard procedures [35,77].

### 4.6. Food Intake Studies

Mice were singly housed and acclimatized in this condition for 2 days. On the day of the experiment, the chow diet was weighed and then placed in the cage at 6 p.m. The remaining amount of food was weighed in the following day at the same time to quantify the 24-h food intake. This process was continued for 7 days. The average amount of food consumed per mouse per day was calculated. The mice had full access to drinking water throughout the study.

### 4.7. Blood Collection and Assessment of Serum Metabolic Parameters

Blood was collected from 4-h-fasted mice by cardiac puncture, and serum was prepared following standard procedure. Serum TAG, NEFA, cholesterol and phospholipids were measured at the Mouse Metabolic Phenotyping Centers (MMPC), University of Cincinnati, College of Medicine Pathology & Laboratory Medicine. Serum insulin concentration was determined by using an ultra-sensitive mouse ELISA kit (Crystal Chem, Elk Grove Village, IL, USA). Serum AST and ALT levels were measured using commercial kits from Teco Diagnostics (Anaheim, CA, USA). Hepatic lipid extraction for TAG measurement was performed following a standard protocol [36].

### 4.8. RNA Isolation and qRT-PCR Studies

RNA isolation and qRT-PCR were conducted following a standard ΔΔCT method [36]. Hypoxanthine guanine phosphoribosyl transferase (*Hprt1*) and acidic ribosomal protein large P0 (*Arp*) mRNA were used as controls for adipose tissue and liver, respectively. The comparative threshold cycle method was used to calculate changes in mRNA abundance.

### 4.9. Histology

Adipose tissue and right lobe of the liver tissue were fixed in 10% neutral buffered formalin for two days. Eight micron-sections were prepared and subsequently stained with hematoxylin and eosin (H&E) [36,66].

### 4.10. Gel Electrophoresis and Immunoblotting

For immunoblotting, total protein was isolated by standard protein lysis RIPA buffer containing the protease-phosphatase inhibitor tablet and quantified using a BCA protein assay kit (Thermo-Scientific, Waltham, MA, USA). An equal amount of total protein was loaded onto 10% SDS-PAGE. Proteins were detected by immunoblotting following our standard protocol [66]. Densitometric analyses of protein bands were performed using the ImageJ software (Java 1.8.0_172).

### 4.11. Statistics

For multiple comparisons, two-way Anova with Holm–Sidak multiple comparison test was used. For two independent data sets, two-tailed Student’s *t*-test was used. Data are presented as mean ± SEM (**** *p* < 0.0001, *** *p* < 0.001, ** *p* < 0.01 and * *p* < 0.05). Statistical significance and area under curve (AUC) were calculated in GraphPad Prism software, v. 7. Immunoblots were quantified using ‘ImageJ’ software. Data are plotted for individual animal to show number of animals per experiment.

## Data Availability

All data are included in the manuscript figures.

## Supplementary Materials

The following supporting information can be downloaded at: https://www.mdpi.com/article/10.3390/ijms23042059/s1.
