# Supplementary material for: Whole Body Ip6k1 Deletion Protects Mice from Age-Induced Weight Gain, Insulin Resistance and Metabolic Dysfunction
Source: Int J Mol Sci. 2022 Feb 12;23(4):2059. doi: 10.3390/ijms23042059 (PMC8878859; doi:10.3390/ijms23042059)
Supplement: Supplementary file 1 [file ijms-23-02059-s001.zip › ijms-1534527-supplementary.pdf]

## Supplementary figures

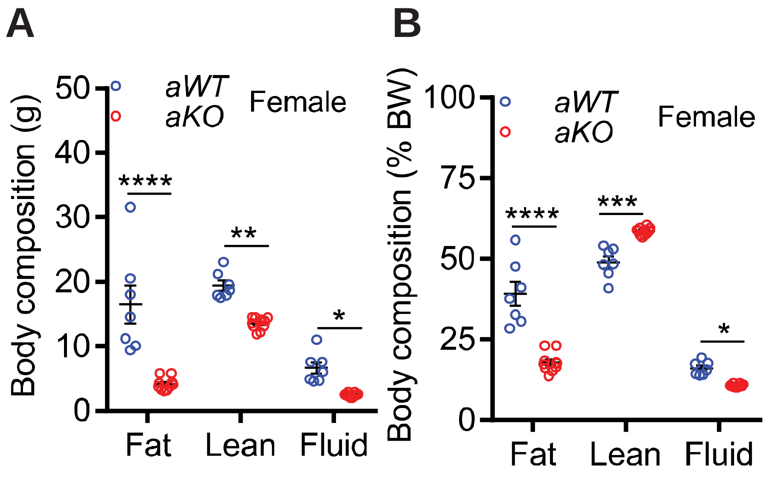


**Figure S1. Whole body deletion of *Ip6k1* protected mice from age-induced weight and fat gain**

**(A)**. Female *aKO* exhibited decreased mass of total fat, lean and fluid compared to *aWT* mice. The analysis was performed in 22-month-old mice. N=7 and 10 for *aWT* and *aKO* mice.

**(B)**. Percent body fat mass was substantially reduced whereas percent lean mass was increased in *aKO* female mice. Percent fluid mass was marginally decreased in *aKO* female mice. N=7 and 10 for *aWT* and *aKO* mice.

Number of mice (*n*) used in each experiment are presented as individual datapoints. Mean ± s.e.m. values are shown within dot plots. For 2 independent data sets, Two-tailed unpaired Student's *t*-test was used. $^{*}P < 0.05$, $^{**}P < 0.01$, $^{****}P < 0.0001$.

**Figure S2. *aKO* mice displayed improved serum metabolic profiles**

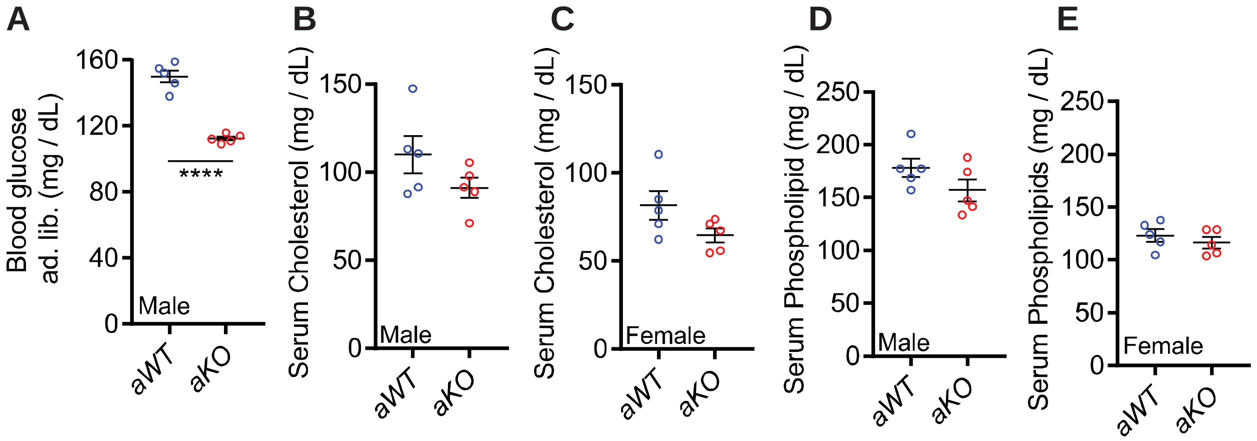


**(A)**. Ad libitum blood glucose level was lower in *aKO* compared to *aWT* mice. N=5 mice each group.

**(B-E)**. Serum cholesterol and phospholipid levels were similar in *aKO* and *aWT* male mice. Female mice also showed similar profiles. N=5 mice each group.

Number of mice (*n*) used in each experiment are presented as individual datapoints.

**Figure S3. Assessment of food intake and activity in *aWT* and *aKO* mice**

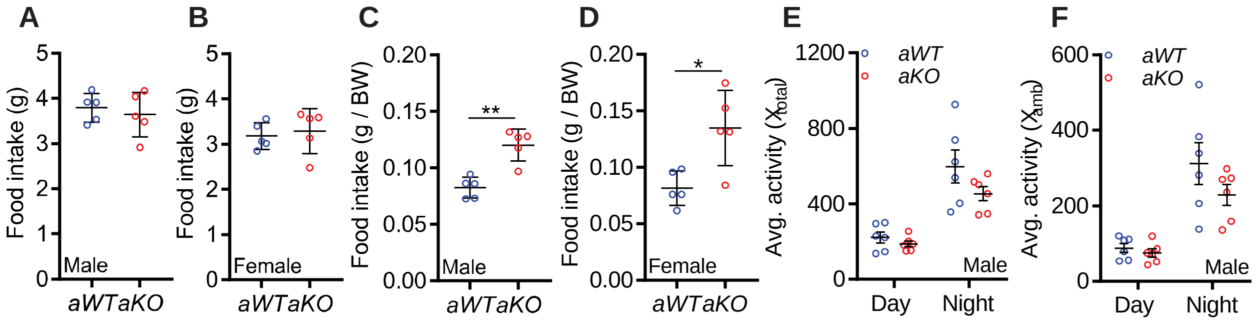


**(A-B)**. *aWT* and *aKO* mice consumed similar amount of food. N=5 mice each group.

**(C-D)**. Food intake / body weight was higher in *aKO* compared to *aWT* mice.

**(E-F)**. Total and ambulatory activity profiles were largely similar in *aWT* and *aKO* male mice. N=6 mice each group.

Number of mice (*n*) used in each experiment are presented as individual datapoints. Mean ± s.e.m. values are shown within dot plots. For 2 independent data sets, Two-tailed unpaired Student's *t*-test was used. $^{*}P < 0.05$, $^{**}P < 0.01$.

**Figure S4. Age-induced metabolic aberration in adipose tissue and liver was ameliorated in *Ip6k1* deleted mice**

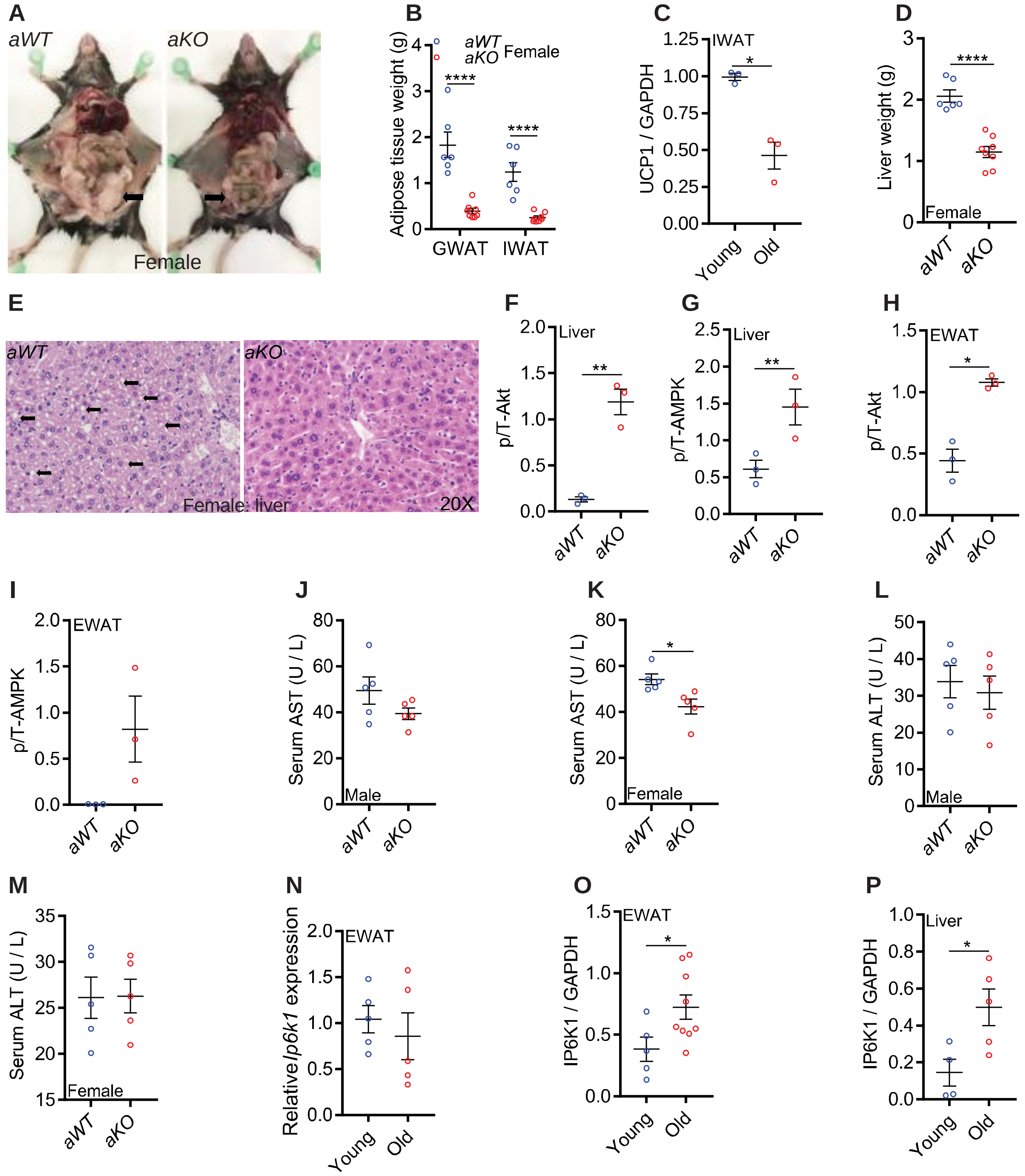

**(A-B)**. *aKO* female mice displayed reduced fat accumulation in adipose tissue depots. Gonadal and inguinal (GWAT and IWAT) adipose tissue depots were smaller in female *aKO* mice. N=6, and 8 for *aWT* and *aKO* mice.

**(C)**. Densitometric analysis of Fig. 4C to quantify UCP1 protein levels in the IWAT of young and old *WT* mice.

**(D)**. Liver weight was substantially lower in *aKO* compared to *aWT* mice. N=6, and 8 for *aWT* and *aKO* mice.

**(E)**. Female *aKO* mice exhibited reduced micro-steatosis in the liver.

**(F-I)**. Densitometric analysis of Figs. 4H-4I to quantify stimulatory phosphorylation levels of Akt (S473) and AMPK (T172) in liver and EWAT of *aWT* and *aKO* mice.

**(J-M)**. Serum levels of AST were insignificantly and significantly reduced in *aKO* compared to *aWT* mice. ALT levels were similar in these conditions. N=5 per group.

**(N)**. mRNA expression of *Ip6k1* was similar in the EWAT of young and old mice. N=5 mice per group.

**(O-P)**. Densitometric analysis of Figs. 4O-4P to quantify IP6K1 protein levels in adipose tissue and liver of young and old mice. GAPDH was used as a loading control.

Number of mice (*n*) used in each experiment are presented as individual datapoints. Mean ± s.e.m. values are shown within dot plots. For 2 independent data sets, Two-tailed unpaired Student's *t*-test was used. $^{*}P < 0.05$, $^{**}P < 0.01$, $^{****}P < 0.0001$.
